# Effects of Tai Chi on depression of middle-aged and older adults: an updated systematic review and meta-analysis

**DOI:** 10.1186/s12906-023-04207-1

**Published:** 2023-10-27

**Authors:** Lijuan Zeng, Xueyang Zhao, Yiqing Yu, Ting Hu, Chaoyang Li, Man Wu, Fen Yang

**Affiliations:** 1 College of Nursing, Hubei University of Chinese Medicine, Wuhan, China; 2 Ningbo Municipal Hospital of Traditional Chinese Medicine (TCM), Affiliated Hospital of Zhejiang Chinese Medical University, Ningbo, China

**Keywords:** Tai Chi, Depression, Meta-analysis, Middle-aged and older adults, Randomized Controlled Trials, Chinese medicine

## Abstract

**Aim:**

The objective of this study was to evaluate the efficacy of Tai Chi, a mind–body movement therapy originating from China, on depression in middle-aged and older adults.

**Methods:**

A systematic search was conducted in seven databases (Embase, Cochrane, Medline, Wanfang, SinoMed, Weipu date, CNKI) for Randomized Controlled Trials (RCTs) published until Apr 16, 2023. The quality assessment, heterogeneity analysis, subgroup analysis, and sensitivity analysis of 12 RCTs selected from the literature were performed. Meta-analyses were conducted using RevMan 5.4 software.

**Results:**

The study included 12 trials comprising 731 participants that met the inclusion criteria. The findings revealed that Tai Chi significantly improved depression in middle-aged and older adults [SMD = -1.21, 95% CI (-1.59, -0.83), I^2^ = 87.6%, *P* < 0.001]. Subgroup analysis revealed that the number of exercise weeks within the specified range, the total duration of exercise, and Tai Chi maneuvers had the greatest benefits on depression in middle-aged and elderly people. The results demonstrated that interventions lasting more than 24 weeks were more effective [SMD = -1.66, 95% CI (-2.28, -1.04), *P* < 0.05] than those lasting only 12 weeks [SMD = -0.73, 95% CI (-1.08, -0.38), *P* < 0.05]. The effect size was more significant when the total duration of the intervention was more than 2400 min [SMD = -1.31, 95% CI (-1.71, -0.92), *P* < 0.001], and when the 24-style Tai Chi exercise was selected [SMD = -1.06, 95% CI (-1.37, -0.75),* P* < 0.001], the difference was also statistically significant. Funnel plots combined with sensitivity analyses, Begg's and Egger's tests indicated no publication bias.

**Conclusion:**

The study suggests that Tai Chi can be an alternative therapy for reducing depression in middle-aged and older adults. It is recommended to prolong the Tai Chi exercise period to more than 24 weeks, with a total exercise duration of more than 2400 min, and 24-style Tai Chi should be selected to achieve the best therapeutic effect in middle-aged and older adults with depression. It should be noted that there may be lower-quality studies in the RCT literature analyzed, which may limit the general applicability and credibility of the conclusions.

## Introduction

Depression is a common mental disease that seriously affects the health of middle-aged and older adults, with a prevalence rate of 2%-32.7%, which is still increasing [1–3]. Depression brings enormous medical costs and economic burdens to patients and society [4, 5], and individuals suffer great pain [6, 7], lower quality of life [1, 8], and shorter life expectancy [9]. It also can present with a depressed mood, loss of interest or pleasure, decreased energy, feelings of guilt or low self-worth, disturbed sleep or appetite, and poor concentration [10]. In addition, the severity of depressive symptoms can fluctuate over time, putting middle-aged and older adults at increased risk of disability and suicide [11, 12]. Depression has emerged as one of the world's most severe and pressing public health problems and the leading cause of disability worldwide [13].

Tai Chi as part of non-pharmacological exercise intervention has a sustained improvement effect on depression. Reviews of non-pharmacological treatments for depression (including Tai Chi, Qigong, yoga, etc.) have reported lower side effects and recurrence rates in middle-aged and older adults and significant reductions in depressive symptoms [14, 15]. This implies that non-pharmacological exercise therapy can be a cost-effective adjunct to antidepressant treatment [14]. As a world intangible cultural heritage, the essential principles of Tai Chi encompass generating internal energy, mind–body integration, mindfulness control of movements and breathing, jing (serenity), and song (loosening) [16]. It is highly appropriate for middle-aged and older adults and has excellent therapeutic effects on depression [17]. One study showed that 28 weeks of Tai Chi practice significantly reduced depression scores and improved quality of life in older women, with the treatment effect, maintained four weeks after cessation of the intervention [18]. Li and many other scholars also declared the positive significance of Tai Chi for mental health, Tai Chi can improve the subjective well-being and depression of middle-aged and older adults [19].

However, the current specific methods of Tai Chi to improve depression in middle-aged and older adults are not uniform, such as exercise cycle [20], total duration [21], and style [22, 23]. And because there are too many factions, the inconsistency in the duration of exercise between different styles of Tai Chi leads to uncertainty about which movement method is most effective. For example, superior effects of modified Chen-Style Tai Chi versus 24-Style Tai Chi on cognitive function, fitness, and balance performance in adults over 55 [22]. Still, some studies have shown that 8-style Tai Chi alone better impacts mental health in middle-aged and older women [23]. Some scholars have even questioned whether Tai Chi can affect depression [24–26]. For example, some scholars agree that Tai Chi positively affects depression [24], but some studies have shown that Tai Chi has no significant impact on reducing the severity of depressive symptoms [25, 26]. At present, there is no systematic review of Tai Chi intervention for depressive symptoms in middle-aged and older adults to elaborate on the specific exercise period, exercise duration, and exercise style. Therefore, this study conducted a meta-analysis to evaluate the effects of Tai Chi intervention on depression in middle-aged and older patients and provided the specific duration and techniques of Tai Chi treatment for depression. Further promoting the wide application of Tai Chi in middle-aged and older adults can improve the depressive symptoms of middle-aged and older adults, reduce the economic burden of society and patients, and promote the development of Tai Chi.

## Method

To enhance our meta-analysis review's potency, clarity, and inclusiveness, we utilized the PRISMA-P guidelines to establish rigorous protocols for our systematic review and meta-analysis [27].

### Search strategy and identification of studies

To gather RCTs on Tai Chi intervention in patients with depression, we thoroughly searched the literature using databases such as Embase, Cochrane Library, Medline, Wanfang, SinoMed, Weipu, and CNKI. Our search terms included "Taichi/tai chi chuan/taichi quan/taijiquan/shadowboxing/taiji/tai chi/t'ai chi chuan" and "Depression/anxiety/depressive disorder/major depression/major depressive disorder/sadness." We enlisted the help of a professional librarian in our search process and used similar titles and keywords related to Depression and Tai Chi. To ensure we captured all relevant articles, we imposed language restrictions in our search and used Boolean terms to maximize our search results. We searched systematically before April 2023.

### Inclusion and exclusion criteria

Inclusion criteria: (a) RCTs published in peer-reviewed journals in either English or Chinese; (b) Study participants were aged 45 years or older; (c) Participants were older adults with a clinical diagnosis of depression or scored above ten on the Geriatric Depression Scale (GDS) or similar depression scales; (d) Tai Chi was used as an intervention, and studies compared Tai Chi with usual care and waitlist control; (e) Outcome measures included at least one of the effects of different exercise durations and/or a measure of psychological well-being.

Exclusion criteria: (a) Participants with Depression associated with other diseases in middle-aged and older adults; (b) Confounding factors in the experimental group were not solely Tai Chi intervention; (c) Participants were placed in or received long-term care in a hospital or nursing home; (d) Participants in the control group received exercise or other physical activity interventions; (e) Unextractable data and/or unresponsive authors to requests for clarification.

### Study selection

Two reviewers (Zeng and Zhao) searched independently using the same data source and search strategy. The retrieved articles from the six databases were imported into Endnote, a reference management software program to help remove duplicates from the final lists, store full texts of the studies and manage references. Then two reviewers (Zeng and Zhao) independently screened titles and abstracts to identify their inclusion eligibility. If discrepancies regarding the eligibility of identified studies occurred between the two reviewers, a third reviewer (Yang) was resolved by discussion.

### Data extraction and quality assessment

The characteristics of the original research were extracted from the data by two reviewers (Zeng and Zhao) independently so that it can reduce potential bias and minimize errors. Similarly, any inconsistencies were resolved through consultation with a third reviewer (Yang). A prearranged table matrix was used to collect and extract relevant information such as the author/s, publication year, follow-up time, participants, depression therapy/intervention, and outcomes design. Additionally, we attempted to contact the authors of an RCT conducted by Lavretsky Helen et al. to request data [28], but we have not received any feedback.

The Cochrane Collaboration tool was used to assess the risk of bias for the included trials [29]. Two reviewers independently assessed the risk of bias for each trial. The methodological quality of the RCTs was evaluated using seven domains, including randomization, allocation concealment, blinding of researchers/participants/assessors, blinding of outcome assessment, incomplete outcome data, reporting of lost participants to follow-up, and other sources of bias. RevMan 5.4 was employed to evaluate the risk of bias.

### Data synthesis analysis

Stata 14.0 was used for all analyses. In total, twelve studies were selected for inclusion in the systematic review. Two statistical tests were performed to evaluate heterogeneity. The Cochrane Q Test (I^2^) and the Chi-Square test (Chi^2^) p-value were used to assess statistical heterogeneity, with studies considered heterogeneous if the p-value was less than 0.10 [30]. Additionally, the percentage of total variation across studies was reported as I-squared (I^2^) (I^2^ = 0%-40%, low heterogeneity, I^2^ = 30%-60%, moderate heterogeneity, I^2^ = 50%-90%, substantial heterogeneity, I^2^ = 75%-100%, considerable heterogeneity) [31].

## Result

### Study selection

**Fig. 1:**
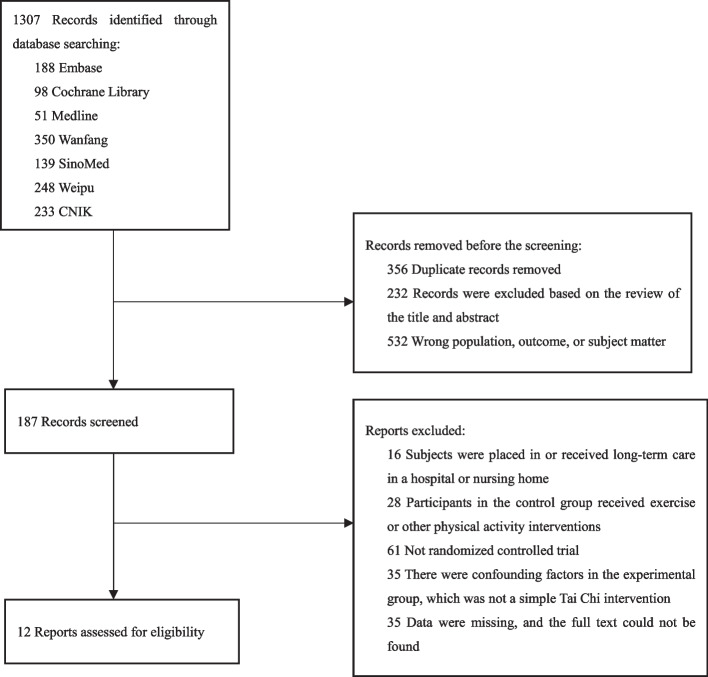
PRISMA selection process summary

The initial database search yielded 1307 citations, of which 356 duplicates were removed. Following the review of the title and abstract, 232 articles were excluded for being irrelevant, while 532 were excluded for involving the wrong population, outcome, or subject matter. Subsequently, 187 documents were evaluated in full text by two independent reviewers, Zeng and Zhao. Among the 187 full-text articles assessed, 175 were excluded because the outcome of interest was not reported (*n* = 35), non-randomized controlled trials were used (*n* = 61), or confounding factors were present in the experimental group, which was not a simple Tai Chi intervention (*n* = 35). Ultimately, the final review included a total of 12 trials [32–43]. A detailed report of the selection process is available in the PRISMA diagram (See Fig. 1).

### Description of studies

Table 1 presents the descriptive characteristics of the 12 studies included in the review. The trials involved 723 participants, all of whom were 45 or older and did not have depression associated with other illnesses.

**Table 1:** Includes the basic information of the literature

study	No. of Participants	N(IG/CG)	Age(y)	Female, No (%)	Assessment tools	Duration, frequency of intervention
Liu 2016 [32]	63	31/32	Range ≥ 65	NA	POMS、SCL-90	16 weeks, 5 times/week, 60 min/time
Xie 2011 [44]	60	30/30	Range ≥ 55	30(50%)	GDS	6 months, 3 times/week, 60 min/time
Lin 2018 [34]	66	33/33	Range ≥ 55	37(56%)	SDS、SAS	8 weeks, 7 times/week, 10 ~ 15 min/time
Li 2020 [35]	60	30/30	Range ≥ 75	36(62%)	GDS-15	3 months, 12 times/month, 40 min/time
Ge 2020 [36]	65	33/32	Range ≥ 60	37(60%)	GDS	8 weeks, 3 times/week, 60 min/time
Li 2011 [37]	74	36/38	Range ≥ 50	48(65%)	POMS-SF	6 months, 5 times/week, 60 min/time
Liu 2018 [38]	60	30/30	Range ≥ 60	32(53%)	GDS	24 weeks, 3 times/week, 60 min/time
Zheng 2021 [39]	60	30/30	Mean (SD),65.32(5.81)	NA	SCL-90	12 weeks, 5 times/ week, 60 min/time
Zhao 2015 [40]	52	26/26	Range ≥ 45	NA	GDS	12 months, 3 times/week, 30 min/time
Liao 2012 [41]	68	35/33	Range ≥ 60	37(54%)	GDS	6 months, 5 times/week, 60 min/time
Fakhari 2017 [42]	56	32/30	Range ≥ 60	NA	BDI-II	12 weeks, 3 times/week, 20 ~ 25 min/time
Yeung 2012 [43]	39	26/13	Range ≥ 55	NA	HAMD-17	12 weeks, 2 times/week, 60 min/times

*Abbreviations*: *IG* Intervention Group, *CG* Control Group, *POMS* Profile of Mood States, *SCL-90* Self-reporting Inventory, *GDS* Geriatric Depression Scale, *SDS* Self-Rating Depression Scale, *SAS* Self-Rating Anxiety Scale, *BDI-II* Beck Depression Inventory-II, *HAMD-17* Hamilton Depression Scale, *NA* Information not available

### Intervention characteristics

The study included 12 articles from 2 countries, with 11 from China and one from the Central American state. The basic characteristics of the included studies are shown in the table. In total, 723 patients were included in this meta-analysis, with 369 assigned to the intervention group and 354 to the control group. Notably, five articles did not provide information on gender [32, 39, 40, 42, 43], while the remaining articles reported 196 male patients and 257 female patients.

All of the studies included in this analysis utilized Tai Chi as an intervention, with seven studies using the 24-style form [32, 34, 36, 37, 40, 41], one study using the 8-style form [35], one study using the 10-step form [42], two studies using both the 24-style and 42-style forms and one study utilizing the 108-style form [33, 38, 43]. Intervention times ranged from 8–52 weeks, with intervention frequency typically occurring three times per week and lasting between 30–60 min.

### Risk of bias

**Fig. 2:**
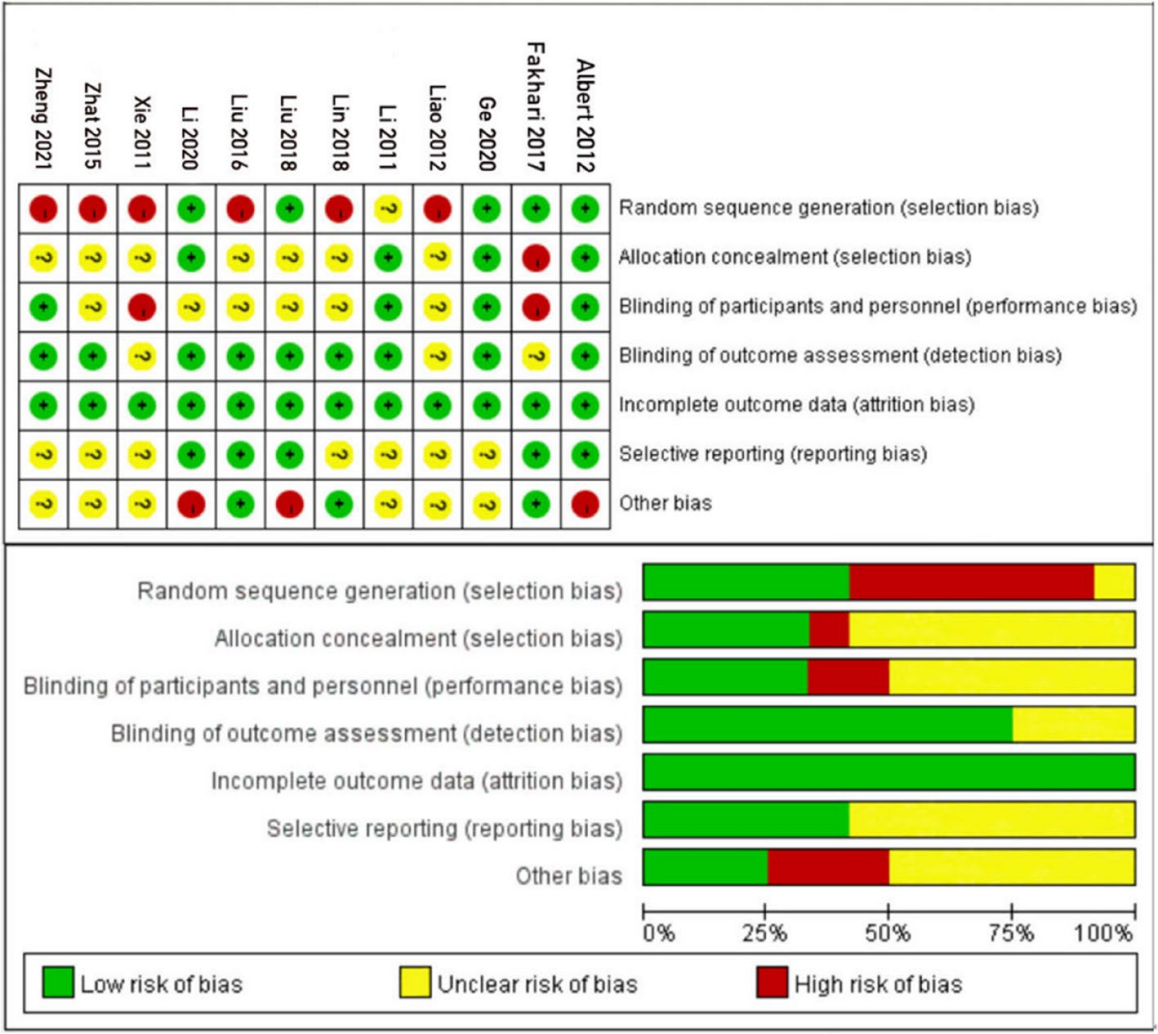
Included literature quality evaluation

The methodological quality of the studies included in this analysis was assessed using RevMan 5.4. A summary of the bias risk assessment chart and the included studies' bias risk summary chart is presented in Fig. 2. Out of the 12 studies analyzed, only five reported the method of generating a random sequence [35, 36, 38, 42, 43], while the other seven studies needed to describe their randomization process clearly. Additionally, only four studies employed participant blinding [36, 37, 39, 43], and nine studies utilized outcome assessors who were blinded to the study's treatment assignment [32, 34–40, 43].

### Meta-analysis

#### Primary outcome

**Fig. 3:**
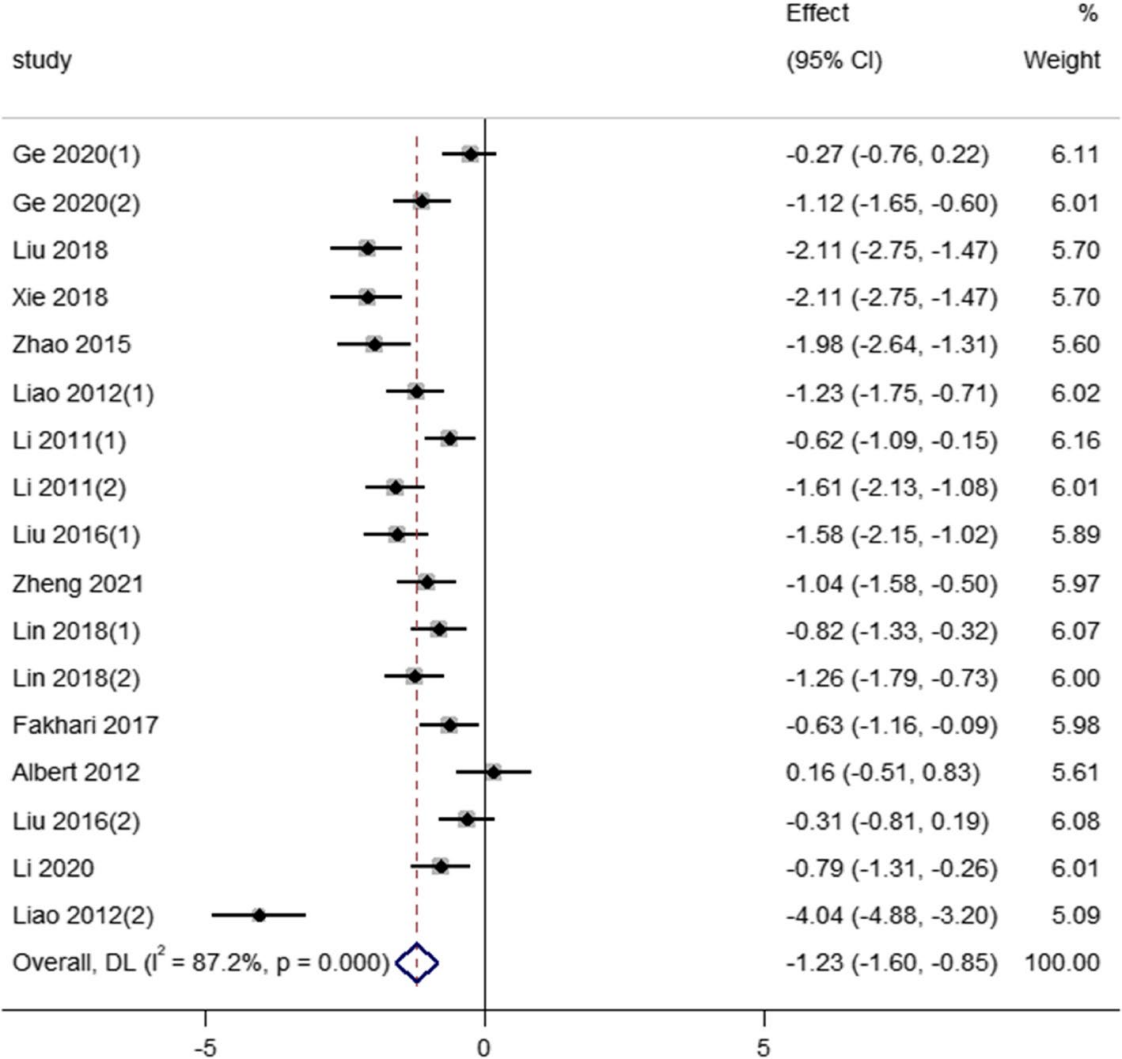
Forest plot of the effect of Tai Chi on middle-aged and older adults depression

Twelve articles were included, and a total of 723 subjects were included, with a total sample size of 369 cases in the experimental group and 354 cases in the control group. The heterogeneity test showed that the heterogeneity was large (I^2^ = 87.2%,* P* < 0.001), and the effect values were combined using a random effect model. The results showed that the depression score of the Tai Chi group was significantly lower than that of the control group [SMD = -1.23, 95% CI (-1.60, -0.85), *P* < 0.001], indicating that Tai Chi intervention training has a significant effect on reducing the depression level of middle-aged and older adults (see Fig. 3).

### Sensitivity analysis

As shown in Fig. 3, significant overall heterogeneity was observed. We performed a sensitivity analysis to address this issue by removing one high-risk study (Liao 2012(2)) and recalculating pooled estimates for the remaining studies. The findings indicated a substantial reduction in heterogeneity [SMD = -1.33, 95% CI (-1.90, -0.76), I^2^ = 80.2%, *P* < 0.001], which remained statistically significant. This study may be because the GDS-30 scale was scored in a way that was not consistent with other literature, leading to its high.

### Subgroup analysis

The number of exercise weeks and total exercise duration of the intervention significantly affected the effect size.

**Fig. 4:**
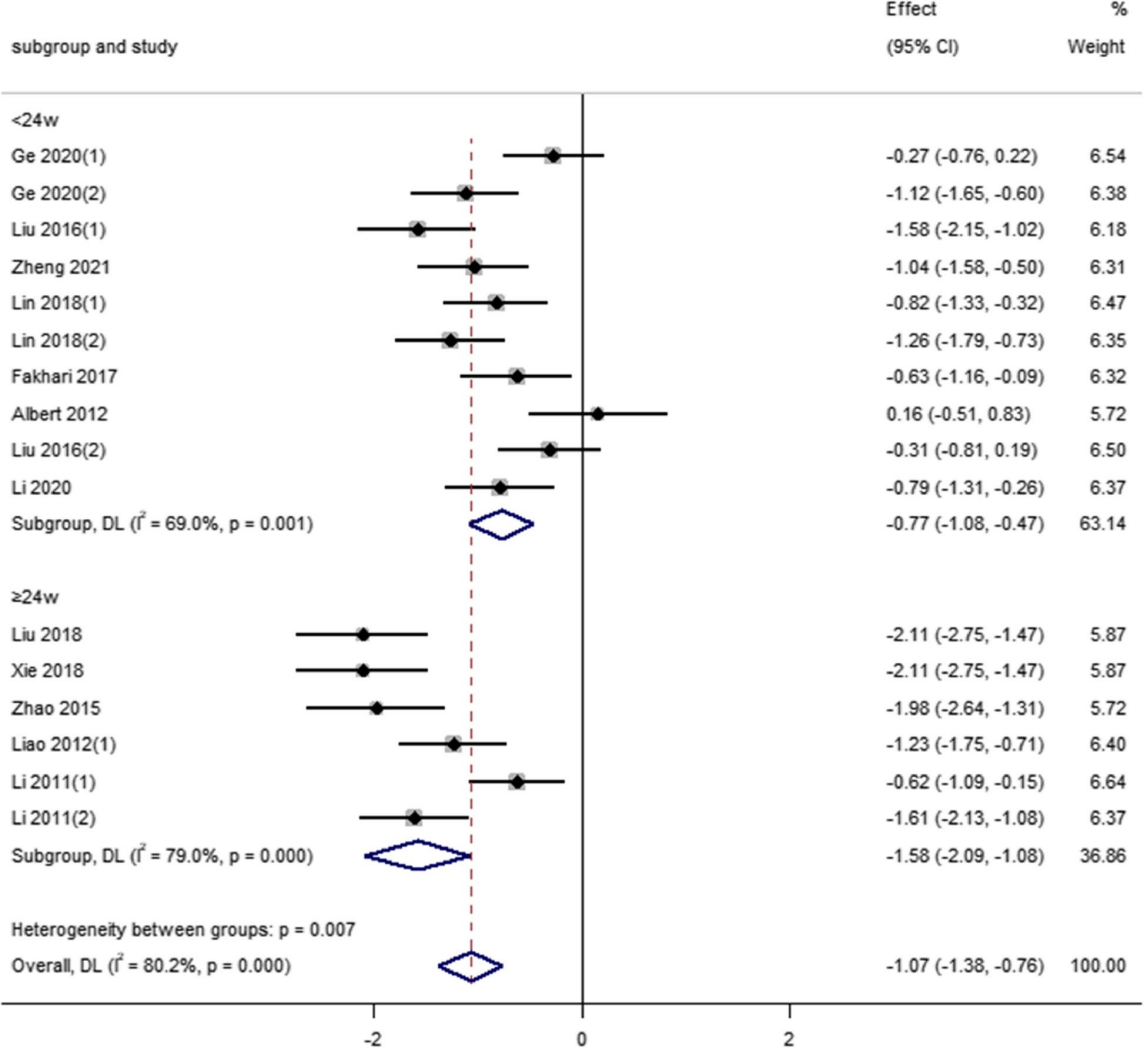
Effect of weeks of Tai Chi exercise on depression index

For the exercise weeks of Tai Chi exercise, among the 12 studies included in depression indicators, the combined effect of seven studies with an intervention period of < 24 weeks as follows [SMD = -0.77, 95% CI (-1.08 to -0.47), *P* = 0.001]. The combined effect of five studies with an intervention period of ≥ 24 weeks was as follows [SMD = -1.58, 95% CI (-2.09, -1.08), *P* < 0.001]. Compared with the control group, the effects of Tai Chi in the two intervention cycles on depression had a significant statistical difference, and there was a statistical difference between the groups (*P* < 0.05), that is, the intervention period of more than 24 weeks could significantly reduce the depression level of middle-aged and older adults (See Fig. 4).

**Fig. 5:**
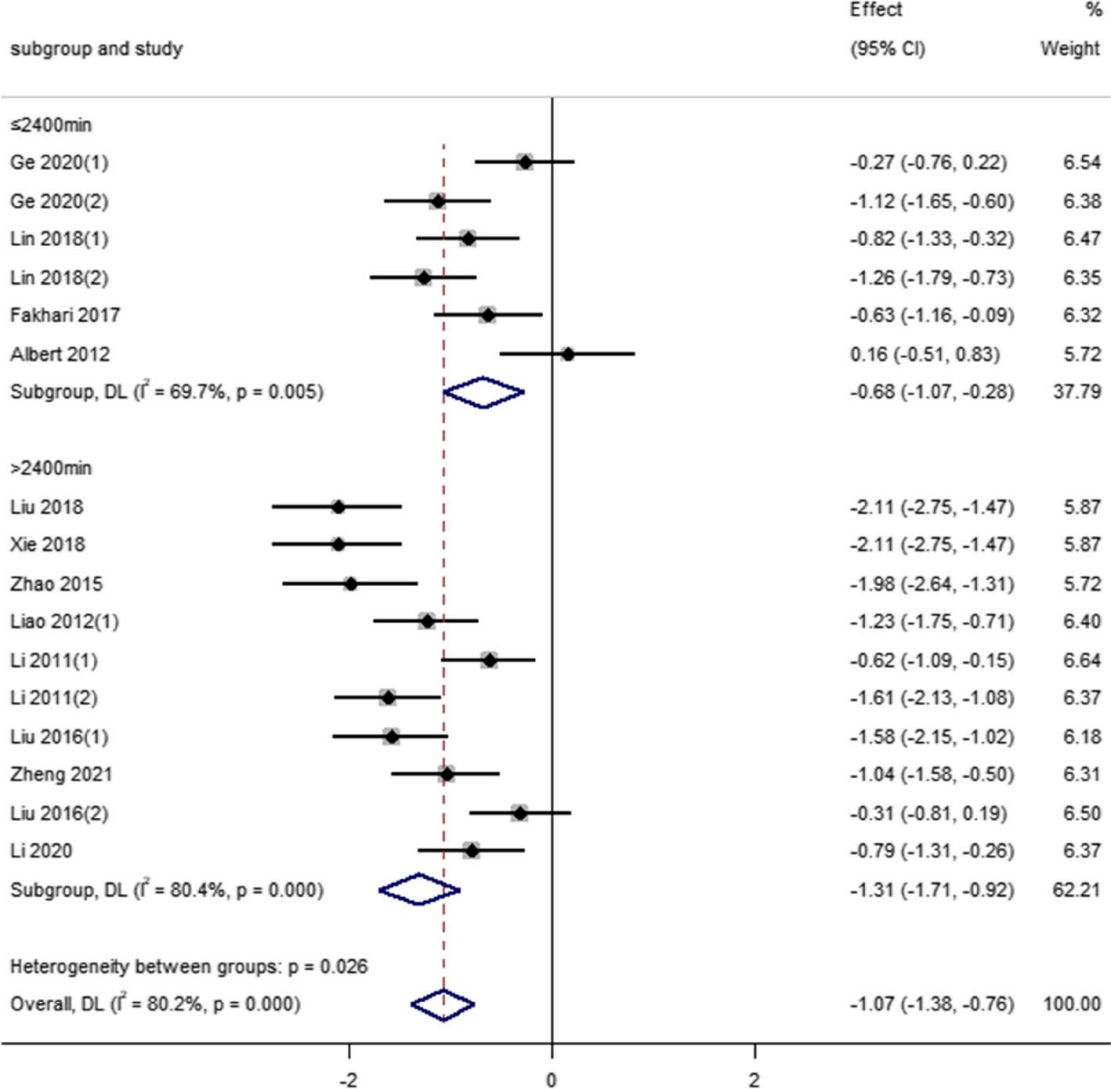
Effect of the total duration of Tai Chi exercise on depression index

For the total exercise duration of Tai Chi exercise, among the 12 studies included in depression indicators, the combined effect of four studies with an intervention period of ≤ 2400 min as follows [SMD = -0.68, 95% CI (-1.07 to -0.28), *P* = 0.005]. For eight studies that exceeded 2400 min, the combined value of the study effect of the intervention cycle of the period was as follows [SMD = -1.31, 95% CI (-1.71, -0.92), *P* < 0.001]. As can be seen, there was a low effect when the total duration of exercise was ≤ 2400 min, while the effect size was significantly larger when the total duration was > 2400 min. Therefore, when the total duration of exercise was > 2400 min, Tai Chi had a greater positive effect on depression in middle-aged and older adults (See Fig. 5).

**Fig. 6:**
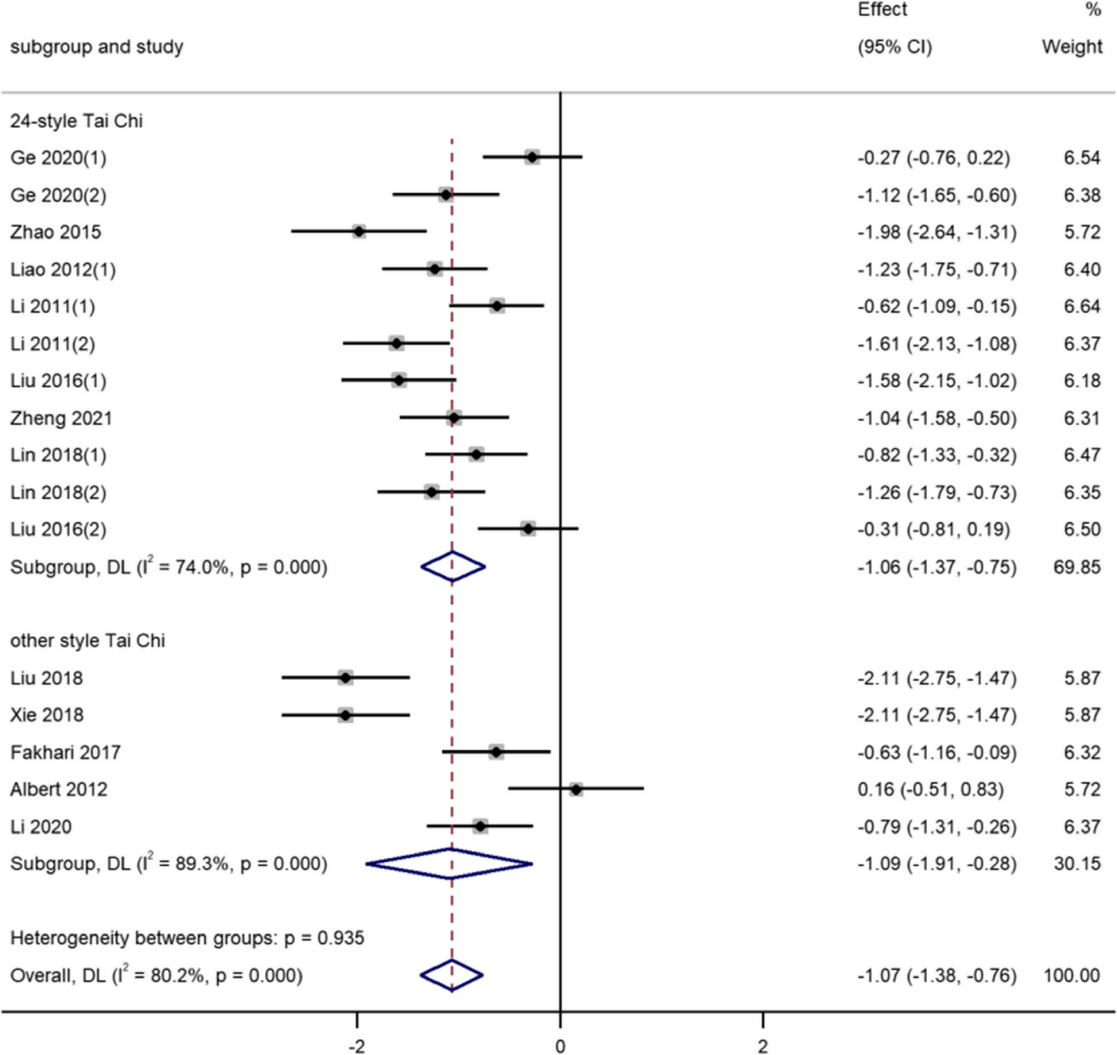
Effects of Tai Chi exercise style on depression index

For the type of Tai Chi, In the course of the 12 studies, there are 8-style form, 10-step form, 12-style form, 24-style form, 42-style and 108-style form. The results showed that the 24-style Tai Chi was more positive for middle-aged and older adults [SMD = -1.06, 95% CI (-1.37, -0.75), *P* < 0.001]. There was no significant difference in other types (See Fig. 6).

### Publication bias

**Fig. 7:**
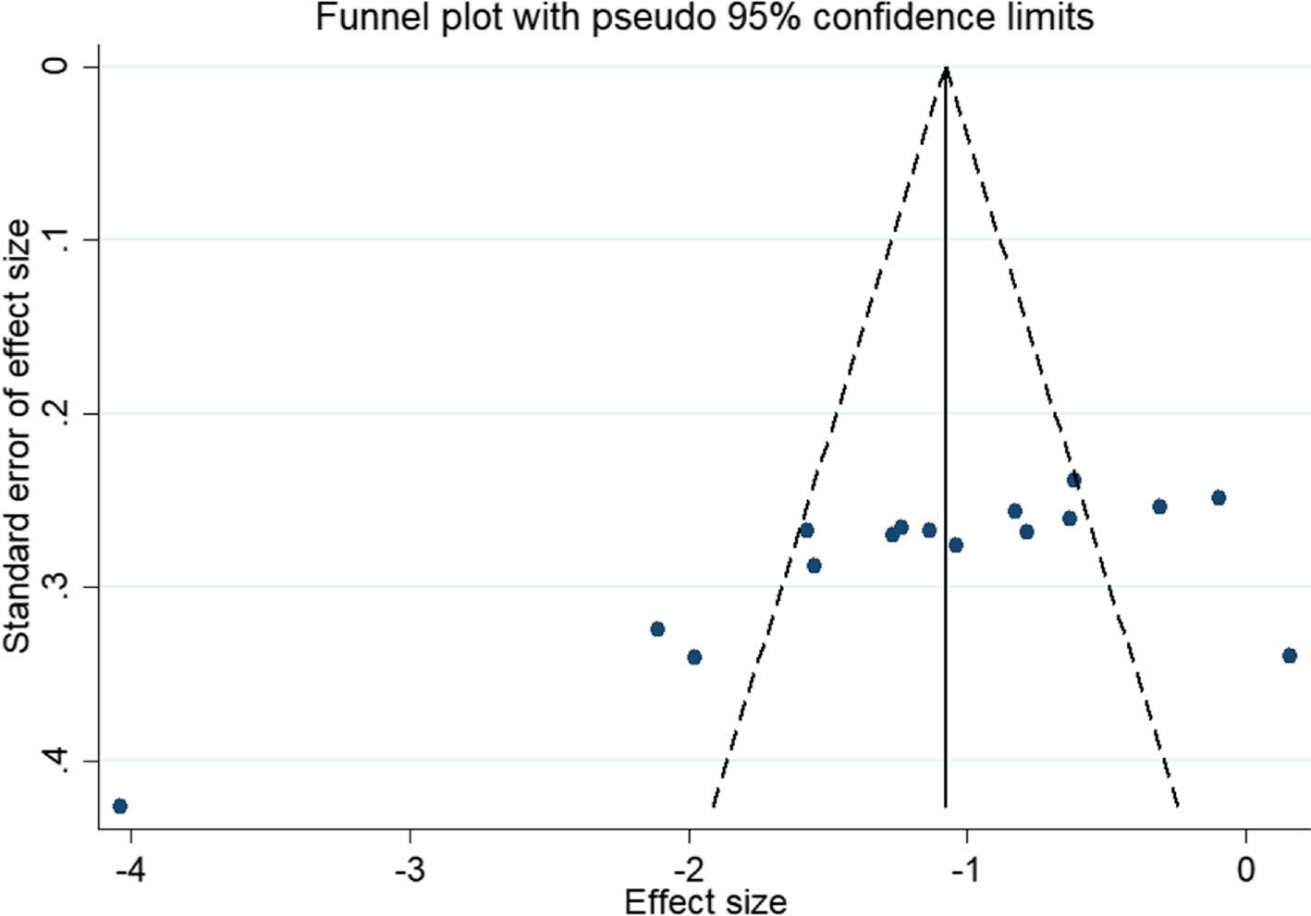
Depression index funnel plot

A funnel plot combined with Begg's test was used to evaluate publication bias. The funnel plot results show that the distribution of studies is uneven and symmetrical, indicating that the included studies may be published, as shown in Fig. 7, Begg test (*P* = 0.059), since *P* > 0.05 suggests there is no obvious publication bias. Otherwise, there is publication bias. The possibility of publication bias is small. The results showed that there was no publication bias for depression.

## Discussion

Currently, with the increasing application prospect of Complementary and Alternative Medicine (CAM) in the management of chronic diseases, non-pharmaceutical treatments are becoming more and more popular among patients with chronic diseases around the world [45]. So far, as a type of CAM, an increasing number of studies have been devoted to exploring the health-promoting effects of Tai Chi on depression, but they have been limited to the duration of individual exercises, total duration, or Tai Chi forms [46–49]. Specific recommendations on how to reduce depression through Tai Chi in middle-aged and older adults remain unknown. Therefore, this meta-analysis analyzed the effect of Tai Chi exercise on improving depressive symptoms in middle-aged and older adults. The study entry points were the overall number of weeks, the total duration of Tai Chi exercise and Tai Chi forms.

In terms of exercise weeks, compared with the control group, the effect of Tai Chi on depression was significantly different in the two intervention periods of less than 24 weeks and ≥ 24 weeks, and the differences between groups were statistically significant (*P* < 0.05). The intervention period over 24 weeks can significantly reduce depression in middle-aged and older adults. The present results are also consistent with previously published studies on the effects of Tai Chi exercise over 24 weeks on improvements in depressive symptoms [50, 51]. This may be because Tai Chi exercise for more than 24 weeks can increase social time and may enhance residents’ interactions with other residents. From the perspective of total exercise duration, duration ≤ 2400 min had a low effect. The study effect size was significantly larger for > 2400 min, suggesting that a certain amount of exercise time may be taken as an essential condition for effect. The results of this analysis further support Yang et al.'s meta-analysis, which declares that the optimal total duration of Tai Chi exercise for depressive symptoms is as follows: more than 1440 min [52].

The difference between the total number of weeks and the total duration of Tai Chi exercise is that by evaluating the total number of weeks of Tai Chi exercise, the degree of stability and long-term adherence of middle-aged and older patients can be understood. Such adherence often contributes to the formation of healthy habits, thereby producing positive physical and psychological effects on depressive symptoms [53]. Again, understand the overall practice time, longer practice time means more physical activity and exercise, which helps to improve heart and lung function, strengthen muscle strength and improve blood circulation, and further improve mood and emotional state [54].

Regarding the type of Tai Chi, 24-style Tai Chi can get better results. This is consistent with the conclusion of Wang and Liu scholars that simplified 24-style Tai Chi is superior to traditional Tai Chi [55, 56]. The reason why 24-style Tai Chi is more effective may be the decline of memory and physical function in middle-aged and older adults. 24-style Tai Chi is easier to remember and less physically demanding than other moves such as 42-style Tai Chi. It is easier to form exercise habits and more sustainable development for the future. Therefore, Tai Chi is an effective way to improve the physical function of middle-aged and elderly people and is more suitable for them.

Therefore, the optimal intervention period for treating depressive symptoms in middle-aged and older adults is ≥ 24 weeks with a total duration of over 2400 min, and the choice of 24-style Tai Chi.

However, although the present meta-analysis found positive results, the results were also consistent with the previous published review on the effect of Tai Chi exercise on improving depressive symptoms in middle-aged and older people [54, 57, 58]; but the small sample size of the included trials, inconsistent efficacy indicators and poor quality of the literature may lead to unstable outcomes. And the positive findings should be interpreted conservatively. Our systematic review had some limitations: (1) No grey literature was included in the primary published studies; (2) Limited to Chinese and English; (3) Some potentially included studies were not included due to data extraction and significant information extraction problems. These limitations are mainly due to the limitation of regional culture in promoting and developing Tai Chi, which is still widely used in China. Moreover, the authors of the included English articles are mainly Chinese, which limits the literature language. Some RCTs should have mentioned accurate data, so they were excluded from the study. Therefore, future high-quality RCTs with large sample sizes, multiple methodological issues, and heterogeneity are needed to support our findings.

## Conclusion

This study is consistent with the conclusion of many scientific reports that physical and mental exercises such as Tai Chi have health benefits on the modification of depression in middle-aged and older adults. This is very encouraging, as these interventions are inexpensive, safe, and can be tailored to individual circumstances. The findings of this study should prompt healthcare professionals, especially mental health professionals, to consider Tai Chi as part of the treatment for depression in middle-aged and older adults. The study draws the following conclusions: (1) Tai Chi can significantly improve the symptoms of depression among middle-aged and older adults. It is a supplementary non-drug resource for depression and has excellent promotion value; (2) There is a precise focus on depression. The intervention period is more than 24 weeks, the total practice time is often more than 2400 min, and the 24-style Tai Chi exercise was used.

## Data Availability

All data generated during this study are included in this published article.
